# Accumulation of advanced glycation end (AGEs) products in intensive care patients: an observational, prospective study

**DOI:** 10.1186/1472-6890-10-4

**Published:** 2010-05-25

**Authors:** Wendela L Greven, Jitty M Smit, Johannes H Rommes, Peter E Spronk

**Affiliations:** 1 Department of Intensive Care Medicine, Gelre hospitals, location Lukas, Apeldoorn, the Netherlands; 2 Department of Internal Medicine, Gelre hospitals, location Lukas, Apeldoorn, the Netherlands; 3 Department of Intensive Care Medicine, Academic Medical Center, Amsterdam, the Netherlands; 4 HERMES critical care group, Academic Medical Center, Amsterdam, the Netherlands

## Abstract

**Background:**

Oxidative stress plays an important role in the course and eventual outcome in a majority of patients admitted to the intensive care unit (ICU). Markers to estimate oxidative stress are not readily available in a clinical setting. AGEs accumulation has been merely described in chronic conditions, but can also occur acutely due to oxidative stress. Since AGEs have emerged to be stable end products, these can be a marker of oxidative stress. Skin autofluorescence (AF) is a validated marker of tissue content of AGEs. We hypothesized that AGEs accumulate acutely in ICU patients.

**Methods:**

We performed an observational prospective study in a medical surgical ICU in a university affiliated teaching hospital. All consecutively admitted ICU patients in a 2 month period were included. Skin AF was measured using an AGE reader in 35 consecutive ICU patients > 18 yrs. As a comparison, historical data of a control group (n = 231) were used. These were also used to calculate age-adjusted AF-levels (AF_adj_). Values are expressed as median and interquartile range [P_25_-P_75_]. Differences between groups were tested by non parametric tests. P < 0.05 was considered statistically significant.

**Results:**

AF_adj _values were higher in ICU patients (0.33 [0.00 - 0.68]) than in controls (-0.07 [-0.29 - 0.24]; P < 0.001). No differences in skin AF_adj _were observed between acute or planned admissions, or presence of sepsis, nor was skin AF_adj _related to severity of disease as estimated by APACHE-II score, length of ICU, hospital stay or mortality.

**Conclusion:**

Acute AGE accumulation in ICU patients was shown in this study, although group size was small. This can possibly reflect oxidative stress in ICU patients. Further studies should reveal whether AGE-accumulation will be a useful parameter in ICU patients and whether skin AF has a predictive value for outcome, which was not shown in this small study.

## Background

Patients admitted to the intensive care unit (ICU) may develop multiple organ dysfunction syndrome (MODS). The pathogenesis of this deterioration of organ function involves oxidative stress caused by reactive oxygen species (ROS) [1]. Indeed, the prognosis and outcome of patients admitted to the ICU is dependent on the degree of oxidative stress [2]. Oxidative stress is associated with most conditions requiring intensive care: but how could oxidative stress be measured? Interestingly, no common strategy exists about its measurement and many methods of estimating oxidative stress have proven unreliable [3]. Moreover most parameters need complicated blood sample measurements.

Advanced glycation endproducts (AGEs) accumulate with oxidative stress and can be very easily and quickly measured, non-invasively. AGEs comprise a diverse class of compounds that link to proteins and accumulate in long-lived tissues. Several AGEs encompass a characteristic autofluorescence pattern when illuminating skin [4]. Using this specific property; skin AGE accumulation can be assessed by the AGE reader, a non-invasive, quick method, that has been thoroughly validated with AGEs measured in skin biopsies [4]. AGEs can be formed by several pathways [5]. In addition to the classical pathway, known as the Maillard reaction, other pathways are important, such as formation by reactive carbonyl compounds, which may form rapidly under oxidative stress [6]. In contrast to most other markers of oxidative stress, AGEs, once formed are stable compounds. They accumulate with age and play an important role in the development of end organ damage in several conditions such as diabetes, atherosclerosis and renal failure [7-9]. Two mechanisms may play a role. First, AGE modification directly alters the function and structure of extracellular matrix proteins [10]. Second, AGEs modulate cellular functions through ligation of specific cell surface receptors, such as the receptor for AGEs (RAGE) [11].

AGE accumulation has been described merely in chronic diseases. But since oxidative stress also augments AGE formation, and oxidative stress occurs in patients in the ICU, we hypothesize an acute rise of AGE levels in patients admitted to the ICU.

## Methods

### Design

This observational study, without interference with usual care was carried out in a university affiliated teaching hospital with an ICU with a mixed medical-surgical closed-format setting with 10 beds. Due to the observational nature of the study without changes in therapy, the requirement for obtaining informed consent was waived by the institutional review board.

### AGE measurements

AGE levels were measured with the AGE-reader, a non-invasive, portable device, which has been thoroughly validated with AGEs measured in skin biopsies [4], DiagnOptics BV, Groningen, the Netherlands. All measurements were performed within 24 hours after admission, consistently by the same observer (WLG). The AGE-reader illuminates a skin surface of 1 cm^2^, with an excitation light source between 300-420 nm. Only light from the skin is measured with a spectrometer in the 300-600 nm range, using 200 um glass fiber. Autofluorescence (AF) will be calculated as follows: ratio between average light intensity, measured in the range 420 - 600 nm and the average light intensity in the range 300-420 nm (emission/excitation) and this ratio is expressed as arbitrary units. Three consecutive measurements, from each patient were taken of which the mean was calculated.

### Patients

Patients were included if skin AF could be measured within 24 hrs after admission. Patients with coloured skin were excluded, because the AGE reader had not yet been validated in patients with coloured skin. Patients with diabetes, liver cirrhosis or renal clearance below 35 ml/min were also excluded, because these conditions are known to cause AGE accumulation.

### Controls

**Figure 1:**
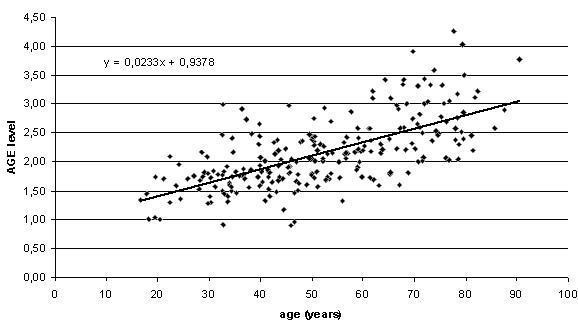
Skin AF levels in control subject show a linear correlation with age, according to an equation: skin AF level = 0.9378 + (0.0233 * age) (R^2 ^= 0.43).

A formerly studied group of healthy volunteers (231 individuals, seen in the outpatient clinic for preoperative evaluation without a history of diabetes, cardiovascular events or renal disease, and all within ASA Class I or II criteria) was used to verify normal AGE levels in healthy individuals (see reference [12] for details)[12]. AGE data were plotted against calendar age to yield a standard skin AF level for each age since AGEs accumulate with age (figure 1). Subsequently, the age-adjusted skin AF (AF_adj_) was calculated according the following formula: skin AF_adj _level = skin AF measured - (0.9378 + (0.0233 * age)) for both patients and controls. After the calculation the skin AF_adj _level for controls approaches zero.

### Statistics

Statistical analyses were performed using SPSS 9.5 for WINDOWS. Non parametric tests were applied. All values are expressed as (median [interquartile range; P_25 _- P_75_]). A P-value < 0.05 was defined to indicate statistical significance.

## Results

### Patients

Thirty-five consecutive ICU patients, 21 male, 14 female were included. The median age in patients was 74 years [57 - 80]. Patient characteristics are shown in table 1. APACHE-II scores as well as length of stay in the ICU were comparable to a one year period preceding this study (data not shown).

**Table 1:** Patient characteristics

	ICU patients
N	35
Age (years)	74 [57 - 80]
Skin AF level	2.81 [2.28 - 3.34]
Skin AF-adj	0.33 [0.00 - 0.68]
ICU LOS (days)	3.00 [2.00 - 6.25]
hospital LOS (days)	13.50 [8.50 - 24.25]
APACHE II	13.0 [10.0 - 16.0]
% died, during ICU-stay	14.3%
% ventilated	94.3%
Admission type	
% acute medical	17.1%
% acute surgical	40.0%
% planned	42.9%
First 24 hrs of ICU stay	
highest glucose level (mmol/l)	8.5 [6.5-11.9]
lowest pH	7.41 [7.36-7.44]
lowest albumin (g/l)	22 [15-24]

LOS = length of stay (days)

Values are expressed as median and IQR [P_25 _- P_75_] or percentages

### Skin autofluorescence measurements

**Figure 2:**
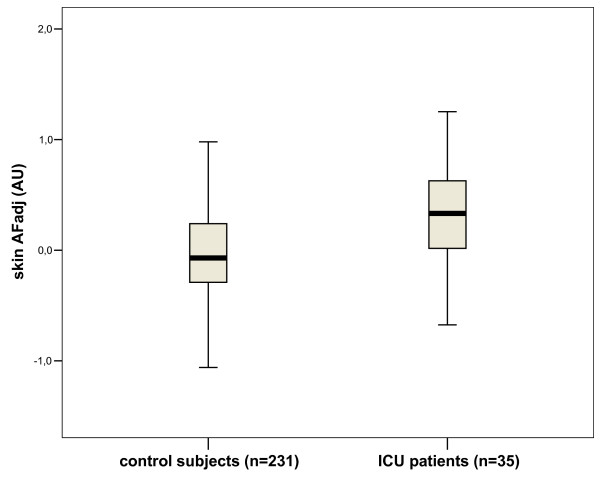
**Boxplots with median, P_25 _and P_75 _The whiskers indicate P_95 _and P_5 _of skin AF adjusted for age (AF_adj_) in controls and ICU patients**. AU = arbitrary units.

Absolute skin AF levels were higher in ICU patients (2.81 [2.28-3.34]) as compared to controls (2.07 [1.74-2.47]; P < 0.001). However, ICU patients were older (74 [57-80 years]) than control subjects (51 [39-67 years]; P < 0.001). In view of the established relation between AGE-levels and calendar age, all further analyses were based on skin AF_adj _(adjusted for age). After the correction for age, skin AF_adj _was still higher in patients admitted to the ICU (0.33 [0.00 - 0.68]) than in controls (-0.07 [-0.29 - 0.24]; P < 0.001) (figure 2).

### Associations with other parameters

No associations were found between skin AF-adj and disease severity as estimated by APACHE-II scores (R = 0.068), ICU length of stay (R = 0.004|), mortality (p = 0.905), nor with sepsis during ICU stay (p = 0.18), the reason for ICU admission (p = 0.544) or the urgency of admission (p = 0.292). In addition, no associations could be found between skin AF-adj and levels of albumin (R = 0.036), highest glucose level (R = 0.016) or the lowest pH (R = 0.00) in the first 24 hours of ICU stay.

## Discussion

This study shows acute AGE accumulation in ICU patients in a direct way by skin auto fluorescence. In this study the accumulation was not associated with disease severity, ICU length of stay or mortality.

Acute AGE accumulation is most likely mediated by oxidative stress, as the classical Maillard reaction is a slow non-enzymatic reaction. Oxidative stress causes formation of reactive carbonyl compounds, which react with protein to form AGEs [6]. Also blocking nitric oxide activity causes the production of reactive oxygen species [13]. Moreover binding of AGEs to RAGE generates intracellular reactive oxygen species [14,15]. This results in diminished reduced glutathione, and ascorbic acid. Depletion of glutathione leads to reduced glyoxalase-1 recycling and decreased in situ activity. Glyoxalase-1, however, has an important role in reducing the cellular AGE load [16,17]. Hence, an acute event could result in acute oxidative stress in the ICU patient, concomitant AGE accumulation, which itself could cause more oxidative stress, thus potentially causing a vicious cycle with progressive multiple organ failure.

The question is whether AGE accumulation is harmful? At least when evaluating chronic accumulation, AGE levels predict future microvascular and cardiovascular events in diabetic patients better than HbA1c [8,9,18]. Moreover skin AGE accumulation predicts (cardiovascular) mortality in hemodialysis patients and in patients with type 2 diabetes [7,19]. In addition, interventions aiming to modulate AGE accumulation have proved to alleviate end-organ damage in animal models [20]. This may support the idea about AGEs playing a causal and potentially predictive role in end organ damage. In contrast to these chronic accumulation of AGEs, recent research is focussed on the less known acute AGE accumulation. One may hypothesize that this acute AGE accumulation, as seen in our study, may be harmful because of intracellular ROS formation as well as depletion of antioxidant mechanisms [15,16]. In ICU patients, one study showed elevated levels of soluble AGE receptors (sRAGE) at admission in septic patients [21]. Furthermore, application of an extracellular decoy for RAGE ligands, improves survival in mice after induction of sepsis, suggesting that RAGE is a central player in perpetuating the innate immune response [22]. Also, AGEs content measured in pericardial fluids seemed to have a prognostic factor after cardiac surgery [23] and plasma levels of sRAGE was associated with increased severity of lung injury and increased mortality in patients with acute lung injury [24]. In addition, plasma sRAGE levels predicted length of stay in ICU in patients after lung transplantation [25]. This is in concordance with our data finding elevated AGE levels already at admission in comparison to control subjects. However, we were not able to find an association with disease severity, ICU length of stay, nor with mortality. Besides differences in case mix and mortality rate, this difference might be explained by the fact that plasma sRAGE levels are not reflecting actual AGE accumulation, thus yielding different results. We did not measure plasma sRAGE in our patients. More research, with larger numbers of patients, and in defined subgroups, should be carried out to address these issues.

Several study limitations need to be addressed. First, this is a small, single center study. Hence, the results may be quite different in other case-mixes in other ICUs. Secondly, this is a small series of patients. Nevertheless, they are probably a reasonable reflection of admissions to our ICU, because the composition of the patient characteristics did not differ with those in a 1 year period preceding this study. Thirdly, the AGE reader itself has not been formally validated for use in an ICU setting since all previous studies have been done in outclinic patients. One might argue that factors like edema could have influenced the results. Indeed, because water is not autofluorescent, values could be mildly reduced in the presence of substantial edema in the arm that was measured. However, this should be structurally addressed in further studies. Fourth; patients with conditions, known to cause AGE accumulation were not included in this study. So AGE accumulation is therefore most likely caused by the ICU admission itself. But the design of this study was cross-sectional, so skin AF values before ICU admission of these particular patients are unknown. Further studies, should maybe focus on longitudinal skin AF measurements to show acute accumulation with ICU admission in the same patient.

In conclusion, acute AGE accumulation occurs in ICU patients, which probably reflects oxidative stress. The group was too small to allow any conclusions on the possible predictive value of skin AF for prognosis for patients on the ICU. Further studies should reveal whether measurement of AGE-accumulation will be a useful parameter in ICU patients.

## Conclusion

Acute AGE accumulation was shown in this small group of ICU patients, which probably reflects oxidative stress. The group was too small to allow any conclusions on the possible predictive value of skin AF_adj _for outcome. Further studies should reveal whether AGE-accumulation will be a useful parameter in ICU patients.

## Abbreviations

AGEs: advanced glycation end products; AF: autofluorescence; ICU: intensive care unit; RAGE: receptor for advanced glycation end products; LOS: length of stay; AU: arbitrary units; ROS: reactive oxygen species.

## Competing interests

Dr J.M. Smit is one of the founders of DiagnOptics BV, the company that manufactures and markets the AGE reader

The other authors declare that they don't have any competing interests.

This study was not funded. DiagnOptics BV kindly provided the AGE-reader for the measurements.

## Authors' contributions

WLG carried out the measurements, did the analysis and interpretation of the data, and wrote the manuscript

JMS has made substantial contribution to the design and revised the manuscript critically for important intellectual content

JHR has revised the manuscript critically for important intellectual content

PES has made substantial contribution to the design, the analysis of the results and revised the manuscript critically for important intellectual content

All authors read and approved the final manuscript.

## Pre-publication history

The pre-publication history for this paper can be accessed here:

http://www.biomedcentral.com/1472-6890/10/4/prepub
